# Daily-Life Fatigue in Mild to Moderate Hearing Impairment: An Ecological Momentary Assessment Study

**DOI:** 10.1097/AUD.0000000000000888

**Published:** 2020-05-15

**Authors:** Louise A. Burke, Graham Naylor

**Affiliations:** Hearing Sciences (Scottish Section), Division of Clinical Neuroscience, School of Medicine, University of Nottingham, Glasgow, United Kingdom.

## Abstract

**Objectives:**

Previous research has indicated an association between hearing impairment (HI) and daily-life fatigue. However, the temporal and contextual correlates of such fatigue are largely unexplored. The present study used ecological momentary assessment (EMA) to examine (1) whether people with HI are more fatigued than people with normal hearing, (2) whether individuals with HI and normal hearing (NH) show similar diurnal patterns of fatigue, (3) whether people with HI spend less time in challenging listening situations compared with NH controls, and (4) whether more challenging listening situations are associated with more fatigue and whether hearing ability influences any observed association.

**Design:**

After excluding 22 participants with self-reported fatiguing health conditions from analyses, the participant sample consisted of 24 adults with HI and 20 adults with NH, aged 44 to 77 years (M = 65.4, SD = 7.5). Data were collected using smartphones and a commercially available EMA app, which ran the specified EMA protocol for this study. Participants responded to six smartphone surveys per day for two weeks. “In-the-moment” questions asked participants to report on their listening situation and to rate their current level of fatigue (“momentary fatigue”) at quasi-random time points throughout the day. Data were analyzed using multilevel modeling.

**Results:**

Hearing group (HI versus NH) was unrelated to trait, daily, and momentary fatigue; both participants with HI and NH became increasingly fatigued throughout the day and at a similar rate. Challenging listening situations occurred infrequently both for HI and NH groups. Participants with NH were more likely to report that there were people speaking in the background whom they were trying to ignore, but participants with HI were more likely to report a greater number of background speakers. No associations were found between within-person listening situations and momentary fatigue, but person-mean listening activity and conversational status were related to momentary fatigue. Notably, having tinnitus was positively related to momentary fatigue, after controlling for other covariates. Finally, having a fatiguing health condition was a strong predictor of both trait and momentary fatigue.

**Conclusions:**

This is the first study to explore and compare fatigue across HI and NH groups using EMA. Contrary to expectations, the groups showed similar levels and diurnal patterns of fatigue, and fatigue was mostly unrelated to aspects of the listening environment. Between-person differences, although statistically significant, produced small effect sizes and therefore must be accepted cautiously. Issues with group matching, the measurement of fatigue, and perceived hearing-related difficulties among participants with NH are notable limitations. However, this study makes a novel contribution to both EMA and hearing research and demonstrates the importance of screening for fatiguing health conditions. Further research is warranted, particularly with individuals with more severe HI.

## INTRODUCTION

Research with both adults (Hornsby & Kipp 2016; Alhanbali et al. 2017) and children (Hornsby et al. 2014) has indicated that individuals with hearing impairment (HI) experience more severe fatigue than their peers with normal hearing (NH). Within the work setting, workers with HI are more likely to take sick leave (Kramer et al. 2006), need longer time to recover after work (Nachtegaal et al. 2009), and are more likely to feel too tired to do anything after work (Hetu et al. 1988; Backenroth-Ohsako et al. 2003) compared with their colleagues with NH. Moreover, there is mixed and inconclusive evidence for the benefit of audiological interventions, such as hearing aids (HAs) and cochlear implants, in reducing fatigue (Hornsby 2013; Hornsby & Kipp; Alhanbali et al.; Holman et al. 2019; Holman et al. 2020). Fatigue can have negative implications for psychological and emotional well-being, self-care, safety, cognitive functioning, productivity, and overall quality of life (Hornsby et al. 2016) and is consequently an important focus within hearing research.

Fatigue is a complex construct for which a singular definition does not exist. It is often conceptualized as multidimensional, such that fatigue may be general, physical, emotional, or mental, for example (Hornsby et al. 2016). It may be acute and transient, triggered by a specific task, activity, or situation, or it may be more long-term, persistent “trait” fatigue (Hornsby et al.; Pichora-Fuller et al. 2016). Fatigue may be measured objectively as a performance decrement or subjectively using self-report. Subjective measures may assess *trait* fatigue by asking how fatigued a person usually feels. Note, however, that when asking how fatigued a person feels at any given moment (termed “momentary fatigue” in this article), one cannot determine whether the expressed level of fatigue reflects transient (acute) or persistent (trait) fatigue.

Particularly pertinent within hearing research are the related concepts of listening effort and listening fatigue. Listening effort must be expended when external factors such as background noise, or personal factors such as hearing loss, make hearing difficult (Pichora-Fuller 2016). The sustained effort required to hear and understand may lead to a specific type of fatigue called listening fatigue (Pichora-Fuller et al. 2016). Research has generally found that more challenging listening conditions are related to greater listening effort (e.g., Houben et al. 2013; Kuchinsky et al. 2013). However, there is only partial evidence to suggest that listening effort does indeed lead to listening fatigue (Dwyer et al. 2019) or general fatigue (Alhanbali et al. 2017), and Hornsby (2013) found no association between listening effort and task-related fatigue.

Nevertheless, research suggests that listening effort and fatigue do occur and, in comparison to individuals with NH, those with hearing loss may be particularly susceptible. In a matched-pairs design, Dwyer et al.’s (2019) cochlear implant recipients reported expending more effort in listening situations and more instances of listening fatigue across a 2-week period than their peers with NH, though levels of general fatigue were numerically (but not significantly) higher in the NH group. Likewise, Alhanbali et al. (2017) reported elevated effort and fatigue scores among participants with HI, compared with those with NH. In a qualitative example, many of the participants with HI interviewed in Holman et al’s (2019) study experienced fatigue, which was attributed to effortful listening and the emotional difficulties associated with hearing loss. Notably, Vas et al. (2017) reported that listening and speaking in conversational situations, and understanding speech in noise, are challenging for individuals with HI. This raises the question if HI individuals may avoid social, conversational situations to avoid or minimize effort and fatigue. Limited evidence suggests that people with HI do avoid challenging social situations (e.g., Lucas et al. 2018; Mikkola et al. 2016; Holman et al. 2019).

Listening fatigue research has provided valuable insights into the associations between hearing loss and fatigue. However, it has been predominantly laboratory based, while the very nature of listening fatigue, and indeed more general acute fatigue, suggests that it results from specific demanding situations or tasks (Hornsby et al. 2016), which may not be replicable in the laboratory. Moreover, findings from the wider fatigue literature indicate that both clinical and healthy samples tend to exhibit diurnal (i.e., across the day) patterns of fatigue (e.g., Stone et al. 1997; Curran et al. 2004; Murphy et al. 2008; Powell et al. 2017), highlighting the need for multiple daily measurements. Retrospective and summary reports of fatigue should be avoided, as evidenced by Friedberg and Sohl’s (2008) findings that chronic fatigue patients recalled average weekly fatigue as higher than “momentary” fatigue. In sum, there is support for measuring daily-life fatigue “in the moment” by obtaining ratings of current fatigue level; repeatedly across the day to capture diurnal patterns and fluctuations; and in real-world “everyday” situations and environments.

Ecological momentary assessment (EMA) refers to a range of methodologies, which use intensive sampling to gather real-world, real-time data, thus increasing ecological validity and minimizing recall bias (Shiffman et al. 2008). Recent evidence supports the application of EMA within hearing research and has demonstrated the success with which EMA methods and technologies are adopted by individuals with HI (e.g., Galvez et al. 2012; Henry et al. 2012; Timmer et al. 2017). EMA has been used to successfully measure and classify auditory context and to assess HA outcomes in real-world settings (e.g., Hasan et al. 2014; Wu et al. 2015; Smeds et al. 2018; Timmer et al. 2018; Jensen et al. 2019).

This study used EMA to address the following research questions:

1. Do participants with HI show higher levels of momentary, daily, and/or trait fatigue than controls with NH?
2. Do participants with HI and NH show similar diurnal patterns of momentary fatigue?
3. Compared with participants with NH, do individuals with HI spend less time in challenging listening situations?
4. Do more challenging listening situations elicit greater momentary fatigue, and does hearing ability influence any observed association?

## MATERIALS AND METHODS

This research has received ethical approval from the West of Scotland Research Ethics Committee (18/WS/0007) and the National Health Service of the United Kingdom R&D (GN18EN094).

### Participants and Recruitment

One hundred ninety-four members of the participant pool of Hearing Sciences—Scottish Section of the University of Nottingham were invited to participate. The study was also advertised to the general public using posters displayed in Glasgow Royal Infirmary and by asking participants to pass on flyers to family and friends who might be interested in participating. Seventy-six participants initiated the study. Of those, nine withdrew due to such factors as work commitments, minding grandchildren, upcoming holidays, perceived burden, and difficulty using the smartphone. Sixty-seven participants completed all sessions, of which 63 were members of the participant database and four were members of the general public who responded to advertisement posters or flyers. Data from one participant were excluded from analysis due to self-admitted data fabrication. Twenty-two participants, who responded “yes” to the baseline question “Do you think you have any health condition which causes you to feel tired?”, were excluded post hoc. Although participants were not obliged to disclose the nature of their condition, some volunteered this information, examples of which included depression, rheumatoid arthritis, and fibromyalgia.

The final sample constituted 44 participants (24 male) aged between 46 and 77 years (M = 65.4, SD = 7.5). Twenty-four were classified as HI, characterized as having a better ear average pure-tone threshold across four frequencies (500 Hz, 1 kHz, 2 kHz, and 4 kHz), or or BE4FA for short, of >25 dB HL (M = 35.7 dB HL, SD = 16.1 dB HL). Of those, 66.6% (n = 16) were HA users and 33% (n = 8) experienced tinnitus. HA and non-HA users were similar across baseline factors including age, gender, employment status, typical sleep quality and duration, social activity level, and trait fatigue (see Measures section for details of measurement). This, coupled with inconclusive evidence for the benefit of HAs in reducing fatigue (Holman et al. 2019; Holman et al. 2020), led to the decision to allow the HI group to consist of both HA and non-HA users.

Twenty participants had NH, defined as a BE4FA of ≤25 dB HL and asymmetry ≤5 dB HL (M = 15.4 dB HL, SD = 6.1 dB HL). Tinnitus was experienced by 35% (n = 7) of this group, while one participant used HAs to alleviate tinnitus. Research has drawn links between tinnitus, disturbed sleep, depression, and fatigue (e.g., Alster et al. 1993; Burgos et al. 2005; Holmes & Padgham 2009; Langguth 2011). However, in the present sample, participants with and without tinnitus reported similar levels of sleep quality, sleep duration, and trait fatigue, and as aforementioned, participants suffering from depression were excluded post hoc. Therefore, it was decided to allow tinnitus sufferers who met audiometric NH criteria to be classified as such. Sample characteristics are summarized in Table 1.

**TABLE 1.** Sample and hearing group characteristics and group differences

Characteristic	Entire Sample (N = 44)	HI Group (n = 24)	NH Group (n = 20)	*P*
Age (y)	65.4, 7.5	68.5, 6.5	61.8, 7.2	0.002
Gender				0.507
Male	24 (54.5%)	12 (50%)	12 (60%)	
Female	20 (45.5%)	12 (50%)	8 (40%)	
Employment status				0.028
Not working	29 (69.9%)	20 (83.3%)	9 (45%)	
Work part-time	8 (18.2%)	2 (8.3%)	6 (30%)	
Work full-time	7 (15.9%)	2 (8.3%)	5 (25%)	
BE4FA (dB HL)	26.5, 17.0	35.7, 16.1	15.4, 6.1	0.000
Asymmetry (dB HL)	10.0, 13.0	15.9, 15.2	2.9, 2.3	0.000
Aided				0.000
Yes	17 (38.6%)	16 (66.7%)	1 (5%)	
No	27 (61.4%)	8 (33.3%)	19 (95%)	
Tinnitus				0.908
Yes	15 (34.1%)	8 (33.3%)	7 (35%)	
No	29 (65.9%)	16 (66.7%)	13 (65%)	
Taking medication which causes fatigue				0.662
Yes	3 (6.8%)	2 (8.3%)	1 (5%)	
No	41 (93.2%)	22 (91.7%)	19 (95%)	
Lifestyle factors which cause fatigue				0.488
Yes	5 (11.4%)	2 (8.3%)	3 (15%)	
No	39 (88.6%)	22 (91.7%)	17 (85%)	
Typical sleep duration				0.273
Under 5 h	2 (4.5%)	0 (0%)	2 (10%)	
5–7 h	29 (65.9%)	17 (70.8%)	12 (60%)	
7–9 h	13 (29.5%)	7 (29.2%)	6 (30%)	
Typical sleep quality				0.907
Very well	10 (22.7%)	6 (25%)	4 (20%)	
Well	9 (20.5%)	4 (16.7%)	5 (25%)	
Okay	20 (45.5%)	11 (45.8%)	9 (45%)	
Badly	5 (11.4%)	3 (12.5%)	2 (10%)	
Percentage of prompted surveys responded to	85.1, 15.5	79.8, 18.0	91.4, 8.8	0.009
Percentage of prompted surveys completed	82.1, 17.8	75.8, 20.9	89.7, 8.9	0.006
No. valid unprompted surveys completed	7.4, 8.1	10.0, 9.6	4.4, 4.6	0.021
Trait fatigue (FAS, scale 0–40)	5.9, 5.2	5.7, 5.3	6.1, 5.2	0.785
Social activity (SAL, scale 0–6)	3.3, 1.1	3.5, 1.2	3.1, 1.1	0.308
Hearing handicap (HHIA/E, scale 0–100)	24.6, 24.4	35, 27.7	12.2, 11.2	0.001
Mean momentary fatigue (BFI “right now” item, scale 0–10)	2.2, 1.7	2.0, 1.7	2.4, 1.8	0.474

Categorical variables are presented as n (%); continuous variables are presented as mean, SD.

BE4FA, better ear four-frequency average; BFI, Brief Fatigue Inventory; FAS, Fatigue Assessment Scale; HHIA/E, Handicap Inventory for Adults/the Elderly; HI, hearing impairment; NH, normal hearing; SAL, Social Activity Log.

### Materials

#### EMA Platform •

Lifedata is a commercially available web-based platform, which allows for the creation and delivery of an EMA protocol via a corresponding smartphone app, RealLife Exp (Lifedata LLC 2015).

#### Smartphones •

Fifteen Honor Lite 9 Android smartphones running the software version LLD-L31 8.0.0.138(C432) were used. Phones were fitted with a glass screen protector and a flip case. Notification alerts were both auditory and vibratory.

#### Participant Resources •

Participants received a phone charger, a mains power socket timer (to avoid overcharging phones), help booklets for the smartphone and app, and an activity document. The purpose of this document was to assist participants in responding to a question which appeared on daytime EMA surveys and unprompted surveys; “what are you doing right now?” Response options were short and undetailed so as not to clutter the smartphone screen. Therefore, participants were provided with an accompanying descriptive document both in hardcopy and as a pdf on the phone to expand on each response option.

### Procedure

Data collection ran from November 2018 to June 2019. Each participant made three visits to the department over a 2-week period, during which they received six EMA surveys per day. In their first visit, participants provided informed consent and partook in an ear examination, audiometry, and completed baseline questionnaires. They were then trained to use the smartphone and app and received a thorough description of the EMA schedule, survey types and time range, response windows, and the types of questions to expect. The EMA survey sequence was initiated, and participants were invited to practice by completing an unprompted EMA survey, which was discarded from the final dataset.

Approximately mid-way through the first week of the study, participants were contacted by the researcher either by phone or e-mail to answer any questions that may have arisen. One week after the first visit, participants returned to the department, where they were queried about their experience of using the phone and the app so far, and data were downloaded from the phone to the web app. The third and final visit occurred approximately 2 weeks after the first. This consisted of data download, post-test questionnaires, and a semistructured interview to elicit overall study experience. Participants received £40 compensation, received as £10 at their first visit, £10 at their second visit, and £20 at their final visit.

### EMA Protocol and Schedule

To initiate the EMA survey sequence, participants first responded to a “start-up” block of questions, which asked for demographic information. From then on, the app fired six quasi-random surveys per day for 14 days. The morning survey was fired randomly between 8 am and 10 am with a 3-hour response window, meaning that participants had up to 3 hours to respond before the survey expired. Four identical daytime surveys were fired between 10.30 am and 7.30 pm, with a 30-minute response window for each. Daytime surveys were separated by a time interval of at least 90 minutes. The evening survey was fired randomly between 8.00 pm and 9.30 pm, with a 3-hour response window.

Participants were also able to initiate an unprompted EMA survey at any time. They were asked to do this if they had missed a prompted daytime survey or experienced a fatiguing event, activity, or situation which they would like to report. Unprompted surveys were identical to prompted daytime surveys but were preceded by the question “Why did you decide to self-initiate a survey?”, with response options (1) “I missed a notification” and (2) “I would like to record a fatiguing event/activity/situation”. Selecting option 2 triggered a follow-up question regarding how long it had been since this event/activity/situation had occurred. Response options were (1) “it is on-going”, (2) “just finished”, (3) “less than 1 hour ago”, and (4) “more than 1 hour ago”. Selecting it is on-going triggered a survey that was worded in the present tense, while the three other responses triggered a past-tense survey. There was no limit on the number of unprompted surveys that could be completed.

### Measures

#### Baseline •

Participants were categorized as HI or NH using audiometric results obtained at baseline. Trait fatigue was measured using the Fatigue Assessment Scale (FAS; Michielsen et al. 2003). This scale uses 10 items to measure usual physical and mental fatigue, with responses on a five-point Likert scale ranging from 0 = never to 4 = always. Scores were totaled to obtain a score between 0 and 40, with higher scores indicating higher trait fatigue. An example item is “I feel no desire to do anything”. Social activity was measured by the Social Activity Log (Syrjala et al. 2009), where 15 items assess the frequency of social activity in the past week and month. Mean social activity scores were computed using Syrjala et al.’s guidelines. This value can range from 0 to 6, with higher scores indicating more social activity.

#### EMA Start-Up Block •

Participants initially responded to a set of demographic questions which measured age, gender, employment status, HA use, tinnitus, fatiguing medication use, fatiguing lifestyle factors, and typical sleep quality and duration (Table 2).

**TABLE 2.** EMA start-up block

Variable	Question/Scale	Measurement	Treatment in Analyses
Age	What age are you?	Age in years	Grand-mean centered
Gender	What is your gender?	0 = female	Uncentered
		1 = male	
Employment status	What is your employment status?	1 = work part-time	Reference category: not working, uncentered
		2 = work full-time	
		3 = not working	
Hearing aids	Do you have a hearing aid?	0 = no	Uncentered
		1 = yes	
Health condition	Do you think you have any health condition which may cause you to feel tired?	0 = no	Participants who responded “yes” were excluded from analyses
		1 = yes	
Tinnitus	Do you ever experience tinnitus?	0 = no	Uncentered
		1 = yes	
Medication	Are you currently taking any medication which may cause you to feel tired?	0 = no	Uncentered
		1 = yes	
Lifestyle factors	Are there any lifestyle factors which cause you to feel tired?	0 = no	Uncentered
		1 = yes	
Typical sleep duration	How many hours do you typically sleep per night?	0 = under 5 h	Treated continuously, uncentered
		1 = 5–7 h	
		2 = 7–9 h	
		3 = 9+ h	
Typical sleep duration	How well do you sleep on a typical night?	0 = very well	Treated continuously, uncentered
		1 = well	
		2 = okay	
		3 = badly	
		4 = very badly	

EMA, ecological momentary assessment.

#### EMA Morning Survey •

Each morning, participants responded to questions measuring the previous night’s sleep quality, continuity and duration, and their momentary fatigue (Table 3). Momentary fatigue was measured in all EMA survey types using an adapted version of the first item on the Brief Fatigue Inventory (BFI; Mendoza et al. 1999); “Please rate your fatigue (weariness, tiredness) by selecting the one number that best describes your fatigue right now”. Responses were on a 10-point rating scale, anchored at 0 = no fatigue and 10 = as bad as you can imagine.

**TABLE 3.** EMA morning survey

Variable	Question/Scale	Measurement	Treatment in Analyses
Sleep quality	How did you sleep last night?	0 = very well	Treated continuously, uncentered
		1 = well	
		2 = okay	
		3 = badly	
		4 = very badly	
Sleep continuity	How many times did you wake up last night?	0 = none	Treated continuously, uncentered
		1 = 1–2 times	
		2 = 3–4 times	
		3 = 5+ times	
Sleep duration	For how long did you sleep for last night?	0 = 9+ h	Treated continuously, uncentered
		1 = 7–9 h	
		2 = 5–7 h	
		3 = under 5 h	
Momentary fatigue	Please rate your fatigue (weariness, tiredness) by selecting the one number that best describes your fatigue right now.	0–10 rating scale, with higher scores indicating greater fatigue	Uncentered

EMA, ecological momentary assessment.

#### EMA Daytime Survey (Prompted and Unprompted) •

Daytime surveys measured type of location, listening activity, conversational status, presence of background speech, quantity of background speakers, background noisiness, and momentary fatigue (Table 4). Original listening activity response options (Table 5) were recoded according to the Common Sound Scenarios (COSS) Framework (Wolters et al. 2016). Questions and responses were devised by the authors, with the exception of the background noisiness item, which was adapted from Wu et al. (2015), and the momentary fatigue item (see EMA Morning Survey section). Surveys were date- and time-stamped.

**TABLE 4.** EMA daytime survey

Variable	Question/Scale	Measurement	Treatment in Analyses
Day of the week	Identified from automatic date-stamp on surveys	1 = Monday	Reference category: Sunday, uncentered
		2 = Tuesday	
		3 = Wednesday	
		4 = Thursday	
		5 = Friday	
		6 = Saturday	
		7 = Sunday	
Weekday vs. weekend	Identified from automatic date-stamp on surveys	0 = weekend	Uncentered
		1 = weekday	
Time	Identified from automatic time-stamp on surveys	hh:mm:ss	Rounded to the nearest hour, centered at 8 am
Type of location	Where are you right now?	1 = in my home	Recoded as
		2 = restaurant/bar/café	0 = at home
		3 = outdoors	1 = not at home, person-mean centered
		4 = shops	
		5 = work	
		6 = in transit	
		7 = other	
Listening activity	What are you doing right now?	Original response options*	Recoded as
		coded as	0 = NS activity
		1 = SC only	1 = SC and/or FL activity, person-mean centered
		2 = FL only	
		3 = SC + FL + NS	
		4 = SC + FL	
		5 = FL + NS	
		6 = SC + NS	
		7 = NS only	
Conversational status	How many people are you listening to/speaking with?	1 = none	Treated continuously, person-mean centered
		2 = 1	
		3 = 2–3	
		4 = 4–5	
		5 = 5 or more	
Presence of background speech	Are there other people in the background who you are trying to ignore?	0 = no	Treated continuously, person-mean centered
		1 = yes	
Quantity of background speakers	How many people in the background are you trying to ignore?†	1 = 1	Treated continuously, person-mean centered
		2 = 2–3	
		3 = 4–5	
		4 = more than 5	
Background noise	What is the level of background noise?	1 = quiet, e.g., library	Treated continuously, person-mean centered
		2 = somewhat noisy	
		3 = noisy	
		4 = very noisy, e.g., busy bar or restaurant	
Momentary fatigue	Please rate your fatigue (weariness, tiredness) by selecting the one number that best describes your fatigue right now.	0–10 rating scale, with higher scores indicating greater fatigue	Uncentered

*See Table 5 for original coding of listening activity item.

†This item was only presented if participants responded “yes” to the previous question.

EMA, ecological momentary assessment; FL, focused listening; NS, nonspecific; SC, speech communication.

**TABLE 5.** Original response options and branching associated with type of location and activity items

Question 1. Where Are You Right Now?*	Question 2. What Are You Doing Right Now?	
In my home	Watching/listening to entertainment	
	Conversation	
	Using the internet	
	Reading	
	Eating	
	Household task	
	Other†	
Restaurant/bar/café	Eating/drinking	
	Conversation	
	Using the internet	
	Reading	
	Other†	
Outdoors	Conversation	
	Exercise	
	Eating	
	Using the internet	
	Gardening	
	Reading	
	Other†	
Shops	Browsing	
	Finding items	
	At checkout	
	Conversation	
	Other†	
Work	Manual labour	
	Speaking on the phone	
	Using computer	
	In a meeting	
	With a client/customer/patient	
	Other†	
Question 1. Where Are You Right Now?	Question 2. What Is Your Mode of Transport?	Question 3. What Are You Doing Right Now?
In transit	Car	Reading
	Train Bus	Watching/listening to entertainment
	Plane	Conversation
	Bicycle‡	Using the internet
	Walking‡	Other†
	Other†	

The final location option was “Other” (not shown in the table). When selected, this led to a free text box where participants were asked to briefly describe their current location type and a second free text box to describe their current activity. Participants could select only one location but multiple activity options from the same branch.

*The branch of activity options which was presented to participants was dependent on the type of location selected.

†Selecting the “Other” activity option always led to a free text box where participants were asked to briefly describe the activity.

‡Selecting “Bicycle” or “Walking” did not lead to Question 3, “What are you doing right now?”, as they are considered to be activities.

#### EMA Evening Survey •

The evening survey measured daytime naps, occurrences and causes of daytime fatigue, and daily fatigue (Table 6). Daily fatigue was measured using the nine-item BFI, which assesses both current fatigue level and the extent and effect of fatigue during the prior 24 hours using 0 to 10 rating scales. A prorated mean score was computed when participants responded to at least 50% of scale items. An example item is “Please rate your fatigue (weariness, tiredness) by selecting the one number that best describes your worst level of fatigue during the past 24 hours”.

**TABLE 6.** EMA evening survey

Variable	Question/Scale	Measurement	Treatment
Naps	Did you have any naps today?	0 = no	Uncentered
		1 = yes	
Daily fatigue	Full BFI	0–10 rating scale	Mean score obtained, uncentered
Event fatigue	Has any particular event or activity made you feel fatigued today?	0 = no	Uncentered
		1 = yes	
Description of fatiguing event	Please briefly describe the activity/event which made you feel tired today*	Free text box	Analyzed descriptively

*This item was only presented if participants responded “yes” to the previous question.

BFI, Brief Fatigue Inventory; EMA, ecological momentary assessment.

#### Post-Test •

Perceived hearing handicap was measured using the 25-item Hearing Handicap Inventory for Adults/the Elderly (HHIA/E; Ventry & Weinstein 1982; Newman et al. 1990). Total scores can range from 0 to 100, with higher scores indicating more severe hearing handicap. Participants aged 65 years and older (n = 26) completed the HHIE, while those younger than 65 years (n = 18) completed the HHIA. Eight participants completed the HHIA/E during their third visit, and 36 participants completed and returned a postal version. Nine missing items from six participants were substituted with those participants’ scale means to obtain a full set of scores from which to compute a single hearing handicap score for each participant. In addition, the FAS was re-administered so that baseline and post-test trait fatigue scores could be compared to assess reactivity. Interview data were collated and summarized descriptively.

### Statistical Analysis

Data were analyzed using IBM SPSS statistics version 25. The primary approach to analysis was multilevel modeling, as data were hierarchal. Multilevel modeling is well-suited for analyzing EMA data, which typically violate the assumptions of independence of observations and errors, and are prone to issues of missing data (Shiffman et al. 2008). The hierarchy in the present dataset was measurement occasions (level 1), nested within days (level 2), nested within individuals (level 3). Level 1 variables are those which vary from survey to survey (e.g., background noise level), level 2 variables vary from day to day (e.g., sleep quality on the previous night), and level 3 variables vary from person to person (e.g., age).

Baseline questionnaire scores were grand-mean centered, and hearing group was uncentered. Centering decisions for survey-level predictors are outlined in Tables 3, 4 and 6. Missing data were handled using maximum likelihood estimation. The alpha level was set to α = 0.05 for all analyses. No corrections were made for multiple significance testing because the analyses were considered exploratory. An initial null model was run to determine the proportion of variance in fatigue scores at each level of the hierarchy.

Differences in mean trait fatigue (measured once at baseline by the FAS) between the HI and NH groups were assessed using an independent-samples *t* test, while multilevel models were conducted to examine the relationships between hearing group and daily fatigue (measured each evening by the full BFI), and hearing group and momentary fatigue (measured in each EMA survey by the BFI “right now” item; RQ 1). Main effects of time of day, hearing group, and the Time of Day × Hearing Group interaction on momentary fatigue were assessed using a mixed effects multilevel model; the linear effect of time was modeled first with random intercepts and fixed slopes and subsequently with random intercepts and random slopes (RQ 2).

This study assumed the following situations/environments to generally be more challenging; being outside the home (versus being at home), being in a speech communication and/or focused listening situation (versus nonspecific activity), being in larger (versus smaller) group conversations, trying to ignore people speaking in the background (versus not), trying to ignore larger (versus smaller) groups in the background, and perceiving background noise level to be more noisy (versus more quiet). Chi-square tests were used to compare time spent in challenging versus easier listening situations among HI and NH groups (RQ 3). Where a significant Chi-square statistic was found, standardized residuals (*z* scores) were examined to determine which categories contributed to the significant result. *Z* scores greater than ±1.96 were significant at the *p* = 0.05 level, greater than ±2.58 were significant at the *p* = 0.01 level, and greater than ±3.29 were significant at the *p* = 0.001 level.

Finally, a multilevel model was built to test the fixed effects of predictors on momentary fatigue (RQ 4). All level 1 predictors were split into their between-person (person mean) and within-person (person mean-centered) component parts by calculating each participant’s mean score on a variable (person mean) and subtracting their raw scores from this value (person mean-centered). All potential control variables (age, gender, employment status, HAs, tinnitus, medication, fatiguing lifestyle factors, social activity level, hearing handicap, trait fatigue, day of the week, weekday versus weekend, time of day, sleep quality, sleep duration, and sleep continuity) were first modeled, and only those that significantly related to momentary fatigue were retained.

Predictors were then added to the model one-by-one, starting with level 1 predictors followed by level 3 predictors. Nonsignificant predictors of momentary fatigue were dropped from the model. Intercepts were allowed to vary across individuals and days; however, slopes were fixed due to model convergence issues associated with modeling slopes as random. As a result, it was not appropriate to test the moderating effect of hearing group on the relationship between situational factors (level 1 predictors) and momentary fatigue as originally intended.

## RESULTS

### Qualitative Results

The findings from post-test semistructured interviews were summarized and collated. Participants mostly described the study in positive or neutral terms. There was a general sense that the study was easy to do, with all but one participant reporting that it did not impinge on everyday life. A minority (n = 5) found the experience more negative, describing the study as boring, repetitive, and intrusive. Some participants found the phone cumbersome to carry around, while others reasoned that they would have been carrying their own phone anyway so it made little difference. Most participants found the phone easy to use and auditory alerts easy to hear, unless the phone was too far away, in a different room, or the surrounding environment was too noisy.

Most participants (n = 30) said that the study period had been a typical 2 weeks for them in terms of their level of social activity, although some (n = 5) had participated in more than usual due to holidays, funerals, and family events, while others (n = 9) had participated in less than usual, attributed to illness and recovery, work, and healthcare appointments. Most (n = 30) said that their level of fatigue had been the same as usual during the study period, although factors such as illness, chronic pain/disease flare-up, bad weather, dark evenings, very warm weather, and increased physical and social activity led to greater levels of fatigue than usual for some (n = 9). A minority (n = 5) reported less fatigue, attributed to pleasant weather and less physical activity than usual.

Most (n = 39) felt that six EMA surveys per day was an appropriate and manageable amount, although some thought it was too many (n = 4), and one suggested it was too few. The main reasons given for missing surveys were not hearing the alert or being in a situation where it would have been difficult or inappropriate to respond. Such situations included driving, church, cinema, theatre, playing golf, looking after grandchildren, and work-related activities, such as being in a meeting, on the phone, or doing physical/manual work.

### Descriptive Results

#### Response Rate, Completion Rate, and Missing Data •

Analyses are based on 44 participants, 616 days and 2963 completed surveys. Depending on the time of day at which the EMA sequence was initiated and the day the phone was returned, participants received between 76 and 82, of a maximum 84, prompted surveys. On average, 85.2% (SD = 15.5) of prompted surveys were responded to and 82.1% (SD = 17.8) were completed (both ranging from 20.7% to 100%). A total of 550 (96.2%) morning surveys, 1953 (81%) daytime surveys, and 566 (92%) evening surveys were responded to. All participants responded to surveys for 10 weekdays and 4 weekend days. Response rate was 85.5% on weekdays and 84.8% on weekends.

Prompted survey response rate was unrelated to age (*r* = −0.15, *p* = 0.318), gender (*t*(42) = 0.61, *p* = 0.545), employment status (*F*(2, 41) = 0.53, *p* = 0.594), tinnitus (*t*(42) = −1.78, *p* = 0.083), typical sleep quality (*F*(3, 40) = 0.50, *p* = 0.686), typical sleep duration (*F*(2, 41) = 0.50, *p* = 0.609), trait fatigue (*r* = −0.23, *p* = 0.138), social activity level (*r* = 0.11, *p* = 0.494), perceived hearing handicap (*r* = −0.20, *p* = 0.198), day of the week (χ^2^(6) = 6.10, *p* = 0.412), weekday versus weekend (χ^2^(1) = 0.27, *p* = 0.601), and time of day (B = −0.004, Wald χ^2^(1) = 0.138, *p* = 0.710) but was related to hearing group; participants with HI were 11% less likely to respond than individuals with NH (Table 1).

Participants initiated a total of 1235 unprompted surveys. Instances were considered invalid and deleted if (a) they were completed on the participants’ first day of the study, indicating a possible practice survey, (b) they were incomplete, (c) the reason for initiation was to record a fatiguing event/activity/situation, but fatigue was rated as 0 (no fatigue), or (d) the survey was completed immediately after the prior survey, and the responses were identical. This left 321 valid unprompted surveys. Participants completed an average of 7.4 (SD = 8.1) valid unprompted surveys each (range: 0 to 31).

Most unprompted surveys (n = 276, 86%) were completed to replace missed prompted surveys. These were further assessed before merging with prompted surveys. They were deemed invalid if they were (a) completed more than 2 hours after the missed survey, (b) completed within 2 hours, but after the next completed prompted survey, or (c) completed outside the daytime survey timeframe (10.30 am to 8.00 pm). One hundred twenty unprompted surveys were merged with prompted survey data, constituting 3.3% of the final dataset. Just 45 (14%) of the unprompted surveys were initiated to report a fatiguing event/activity/situation, constituting 10.1% (n = 9) of NH and 15.5% (n = 36) of HI unprompted survey instances. These data were discarded as being too few cases to analyze reliably.

#### Reactivity •

A paired samples *t* test showed no significant difference between FAS scores at baseline (M = 15.9, SD = 5.2) and post-test (M = 16.1, SD = 5.2), *t*(43) = −0.38, *p* = 0.708, suggesting no reactivity to the method.

#### Null Model •

Variance in fatigue scores was attributable to person-to-person differences (57.3%), day-to-day differences (9.8%), and survey-to-survey differences (32.9%).

### RQ 1: Do HI Participants Show Higher Levels of Fatigue Than NH Controls?

There was no significant difference in mean trait fatigue (FAS scores) between the HI (M = 5.7, SD = 5.3) and NH groups (M = 6.1, SD = 5.2), *t*(42) = 0.28, *p* = 0.785, and hearing group did not predict daily fatigue, as measured by the full BFI each evening (*F*(1, 43.99) = 0.77, *p* = 0.386) or momentary fatigue, as measured by the BFI “right now” item at each EMA survey (*F*(1, 43.97) = 0.53, *p* = 0.471). These findings are not explained by differential levels of involvement in social activity between the two groups; there was no difference in mean social activity level between participants with HI (M = 3.5, SD = 1.2) and those with NH (M = 3.1, SD = 1.1), *t*(42) = −1.03, *p* = 0.335 and no interaction between social activity and hearing group on trait fatigue (*F*(1, 40) = 1.20, *p* = 0.280), daily fatigue (*F*(1, 43.94) = 0.38, *p* = 0.539), or momentary fatigue (*F*(1, 43.96) = 0.56, *p* = 0.577). In evening EMA surveys, participants with HI were no more likely to report having napped that day (χ^2^ (1) = 1.23, *p* = 0.267) or that something fatiguing had happened during the day (χ^2^ (1) = 3.44, *p* = 0.064) than controls with NH.

### RQ 2: Do HI and NH Participants Show Similar Diurnal Patterns of Fatigue?

**Fig. 1.**
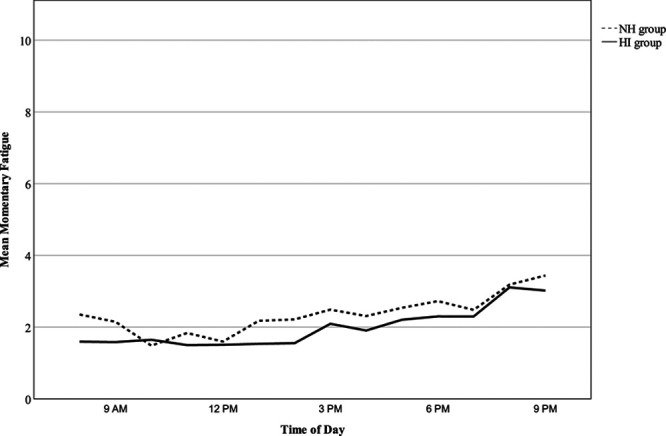
The trajectory of momentary fatigue between the hours of 8 AM and 9 PM on an average study day across hearing groups. The solid line represents the HI (hearing impairment) group and the broken line represents the NH (normal hearing) group.

Time of day significantly predicted momentary fatigue (B = 0.12, *t*(2648.3), *p* < 0.001), reducing residual variance from 32.9% in the null model to 28.8%. As shown in Figure 1, momentary fatigue increased throughout the day: for the group with HI at a mean rate of 0.12 units per hour (*F*(1, 1365.71) = 308.49, *p* < 0.001) and for the group with NH at a rate of 0.11 units per hour (*F*(1, 1282.1) = 210.04, *p* < 0.001). Although diurnal patterns of momentary fatigue were identified, modeling slopes as random showed that the relationship between time of day and momentary fatigue varied significantly across participants (*var*(u_1k_) = 0.008, Wald χ^2^ = 4.02, *p* < 0.001) and days (*var*(u_1j_) = 0.005, Wald χ^2^ = 4.22, *p* < 0.001) and further reduced residual variance to 23.8%. Hearing ability was not predictive of momentary fatigue (B = −0.41, *t*(44.03) = −0.79, *p* = 0.436), and there was no interaction between time of day and hearing ability on momentary fatigue (B = 0.01, *t*(43.68) = 0.17, *p* = 0.867).

### RQ 3: Compared to People With NH, Do HI Individuals Spend Less Time in Challenging Listening Situations?

#### Type of Location •

**Fig. 2.**
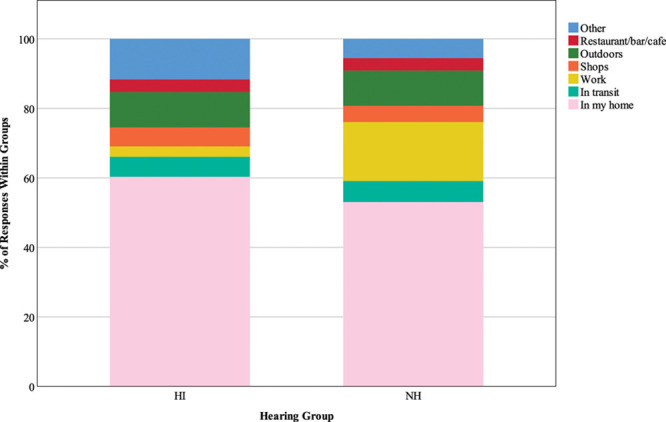
Type of location. The key shows response options for the question “Where are you right now?”, and stacked bars represent the distribution of responses as percentages of 1063 HI responses and 1007 NH responses. HI, hearing impairment; NH, normal hearing.

Figure 2 represents responses to the question “where are you right now?” Both groups most frequently reported being at home (60.3% of HI responses; 53% of NH responses). There were significant differences between the number of expected and observed responses of “work” and “other” locations (χ^2^ (6) = 130.64, *p* < 0.001). Just 3% of HI responses reported being in work (*z* = −7.1), compared with 17% of NH responses (*z* = 7.3). In addition, participants with HI selected the “other” location more often than expected (11.7% of responses; *z* = 3.3), while participants with NH chose this option less often than expected (5.6% of responses; *z* = −3.4).

#### Listening Activity •

**Fig. 3.**
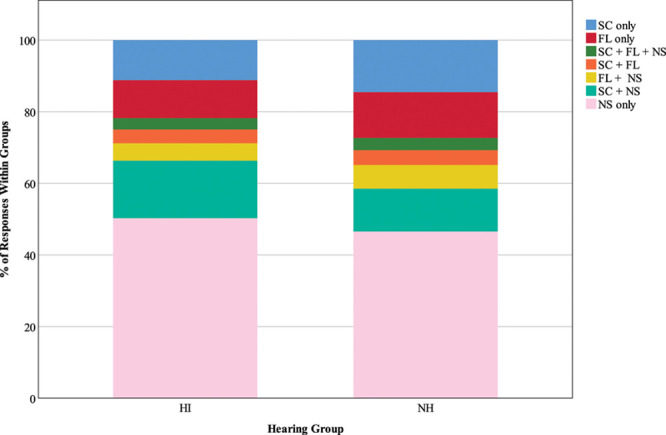
Listening activity, recoded according to the Common Sound Scenarios (COSS) framework. The key shows the categories into which original responses to the question “What are you doing right now?” were recoded. Stacked bars represent the distribution of responses as percentages of 1045 HI responses and 995 NH responses. HI, hearing impairment; FL, focused listening; NH, normal hearing; SC, social communication; NS, nonspecific.

**Fig. 4.**
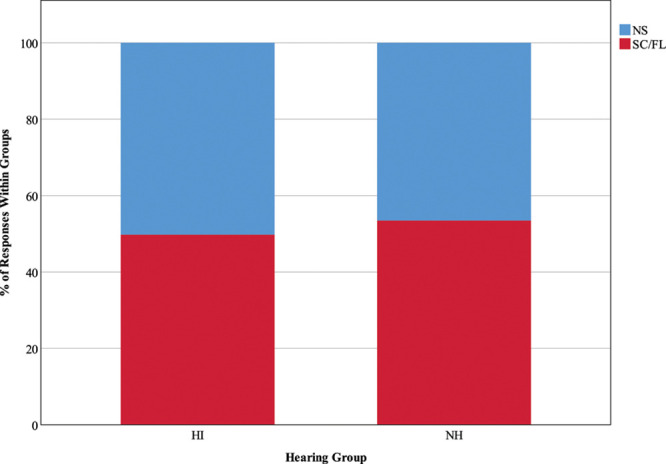
Listening activity, dichotomously recoded as speech communication and/or focused listening (SC/FL) vs. nonspecific activity (NS). HI, hearing impairment; NH, normal hearing.

Figures 3 and 4 show COSS-coded and binary-coded responses, respectively, to the question “what are you doing right now?” The distribution of time spent in each of the COSS-coded listening activities was similar across groups, although statistical results suggest that there are significant differences between expected and observed values (χ^2^ (6) = 17.06, *p* = 0.009). This is most likely driven by the observed 12% of HI responses (less than expected; *z* = −1.8) compared with 16.1% of NH responses (more than expected; *z* = 1.7), indicating participation in social communication and nonspecific activities simultaneously, although note that the corresponding *z* scores have not reached statistical significance. The binary-coded version of the listening activity variable does not reflect these differences; the observed proportion of time spent in speech communication and/or focused listening compared with nonspecific activities was as expected for both groups, χ^2^ (1) = 2.8, *p* = 0.094.

#### Conversational Status •

**Fig. 5.**
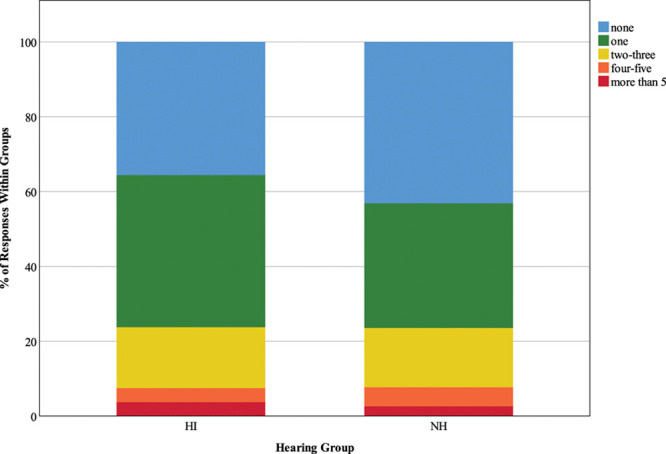
Conversational status. The key shows response options for the question “How many people are you listening to/speaking with?”, and stacked bars represent the distribution of responses as percentages of 1058 HI responses and 1003 NH responses. HI, hearing impairment; NH, normal hearing.

Figure 5 shows responses to the question “how many people are you listening to/speaking with?” Participants in both groups most frequently reported being in nonconversational situations or in one-to-one conversation, with larger group conversations occurring relatively infrequently. A significant Chi-square statistic was found, χ^2^ (4) = 18.92, *p* = 0.001, driven by a greater than expected number of NH reports of not being in conversation (43.2% of responses; *z* = 2.0) and a less than expected number of NH reports of being in one-to-one conversation (33.3% of responses; *z* = −2.0). Meanwhile, the number of HI responses of not being in conversation (35.6% of responses; *z* = −1.9) and of being in one-to-one conversation (40.6% of responses; *z* = 1.9), compared with expected values, were bordering on statistical significance.

#### Presence of Background Speech and Quantity of Background Speakers •

**Fig. 6.**
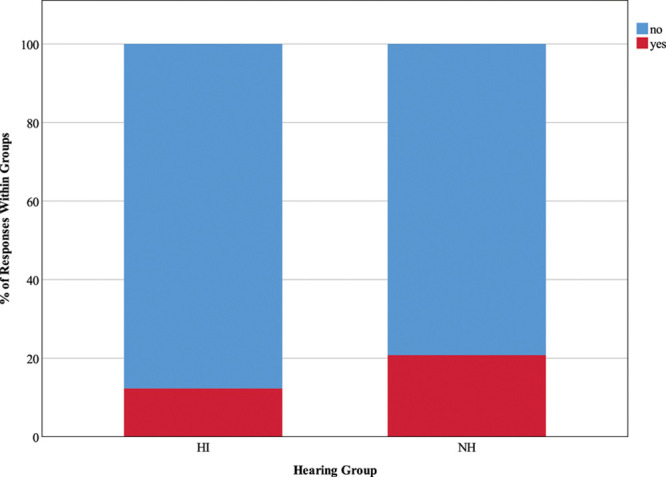
Presence of background speech. The key shows response options for the question “Are there other people in the background you are trying to ignore?”, and stacked bars represent the distribution of responses as percentages of 1059 HI responses and 1003 NH responses. HI, hearing impairment; NH, normal hearing.

**Fig. 7.**
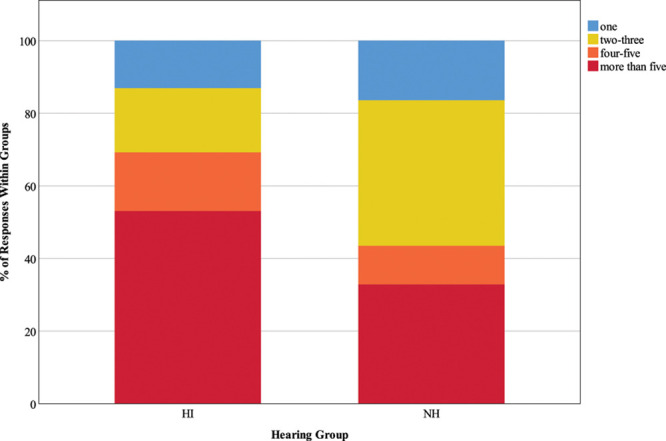
Quantity of background speakers. The key shows responses options for the question “How many people in the background are you trying to ignore?”, and stacked bars represent the distribution of responses as a percentage of 130 HI responses and 270 NH responses. HI, hearing impairment; NH, normal hearing.

Figure 6 illustrates responses to the questions “are there other people in the background who you are trying to ignore?” Only 38.5% of HI responses reported that they were trying to ignore others speaking in the background (less than expected; *z* = −3.3), compared to 61.5% of NH responses (more than expected; *z* = 3.4), and these rates were significant, χ^2^ (1) = 26.91, *p* < 0.001. Figure 7 depicts responses to the question “how many people in the background are you trying to ignore?” A significant Chi-square statistic (χ^2^ (3) = 23.28, *p* < 0.001) was driven by the following findings; HI participants were less likely than expected to report ignoring 2 to 3 people in the background (17.7% of responses; *z* = −2.8) but more likely to report ignoring more than five people (53.1% of responses; *z* = 2.2), while NH individuals spent more time than expected ignoring 2 to 3 people (40.1% of responses; *z* = 2.2).

#### Background Noisiness •

**Fig. 8.**
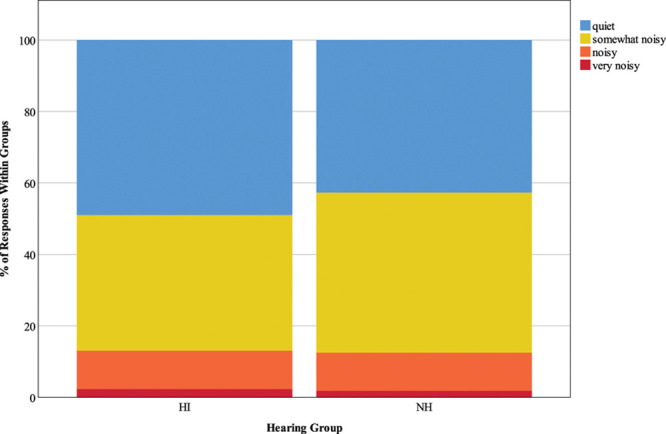
Background noisiness. The key shows responses for the question “What is the level of background noise?”, and stacked bars represent the distribution of responses as a percentage of 1059 HI responses and 1002 NH responses. HI, hearing impairment; NH, normal hearing.

Finally, Figure 8 represents responses to the question “what is the level of background noise?” For both groups, background noisiness was mostly rated as “quiet” or “somewhat noisy”, with very infrequent reports of “noisy” or “very noisy”. Expected and observed responses were significantly different, χ^2^ (3) = 10.85, *p* = 0.013; compared to expected values, participants with HI more frequently rated noise level as “quiet” (49% compared to 42.7% in the NH group), and participants with NH more often than expected rated noise level as “somewhat noisy” (44.8% compared to 38% in the HI group), although *z* scores were nonsignificant.

### RQ 4: Do More Challenging Listening Situations Relate to Momentary Fatigue, and Does Hearing Ability Influence Any Observed Association?

Having controlled for tinnitus, trait fatigue, time of day, and sleep quality and duration on the previous night, level 1 within-person predictors had no effect on momentary fatigue. Effects of individual predictors are presented in Table 7. Reintroduction of level 1 person means as level 3 predictors revealed a significant positive association between person mean listening activity and momentary fatigue, *F*(1, 37.43) = 5.08, *p* = 0.030. This suggests that participants who spent more time in speech and/or listening situations relative to nonspecific activities appear to report higher levels of momentary fatigue. There was also a significant positive association between person mean conversational status and momentary fatigue, *F*(1, 34.34) = 6.04, *p* = 0.019, indicating that participants who spent more time in larger conversational groups, relative to being in conversation with smaller groups or not being in conversation at all, tended to report higher momentary fatigue. Hearing group (coded as 0 = NH, 1 = HI) was not predictive of momentary fatigue, *F*(1, 34.02) = 0.00, *p* = 0.991.

**TABLE 7.** Fixed effects of predictors on momentary fatigue after controlling for covariates

Construct	B	SE	*t*
Controls			
Tinnitus	1.17	0.40	2.94*
GMC trait fatigue	0.20	0.04	5.33†
Time of day	0.11	0.01	21.62†
Sleep quality on the previous night	0.27	0.05	4.88†
Sleep duration on the previous night	0.33	0.09	3.64†
Level 1 predictors			
PMC type of location	−0.00	0.26	−0.00
PMC listening activity	−0.10	0.16	−0.60
PMC conversational status	−0.09	0.06	−1.43
PMC presence of background speech	−3.56	1.89	−1.88
PMC quantity of background speakers	−0.03	0.07	−0.47
PMC background noisiness	−0.08	0.10	−0.80
Level 3 predictors			
PM type of location	−0.39	1.47	−0.26
PM listening activity	2.26	1.00	2.25‡
PM conversational status	1.30	0.53	2.46‡
PM presence of background speech	2.21	1.82	1.21
PM quantity of background speakers	−0.11	0.30	−0.36
PM background noisiness	0.93	0.66	1.42
Hearing group	0.00	0.38	0.01

*p < 0.01;

†*p* < 0.001;

‡*p* < 0.05.

B, unstandardized beta; GMC, grand mean-centered; PM, person mean; PMC, person mean-centered; *t*, *t* test statistic.

### Tinnitus and Fatigue

Although this study did not intend to explore the effect of tinnitus on fatigue, it is worth noting that although tinnitus was unrelated to trait fatigue (*t*(42) = 0.55, *p* = 0.587), it was significantly and positively associated with both momentary (see Table 7) and daily fatigue (B =1.09, *t*(43.86) = 2.43, *p* = 0.019) after controlling for trait fatigue, time of day, and sleep quality and duration on the previous night.

### Health Conditions and Fatigue

**Fig. 9.**
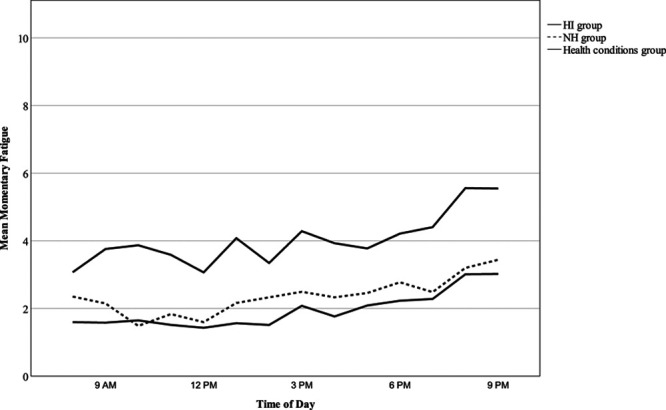
The trajectory of momentary fatigue between the hours of 8 AM and 9 PM on an average study day across HI (n = 24), NH (n = 20), and health conditions groups (n = 22). The solid line represents the HI group, the broken line represents the NH group, and the intermittently solid and broken line represents the health conditions group. HI, hearing impairment; NH, normal hearing.

As aforementioned, data from 22 participants who self-reported a fatiguing health condition were excluded from analyses. This subgroup reported significantly higher trait fatigue (M = 12.6, SD = 7.9) than the 44 healthy participants who constituted the final sample (M = 5.9, SD = 5.2), *t*(30.20) = −3.62, *p* = 0.001. Reporting a fatiguing health condition was also associated with higher momentary fatigue (B = 2.02, *t*(65.95) = 4.23, *p* < 0.001). Figure 9 shows elevated momentary fatigue at all time points across the day among the health conditions group compared to the HI and NH groups. These results illustrate the importance of screening for fatiguing health conditions and potentially excluding participants on this basis to gain an accurate measurement of fatigue in hearing research.

## DISCUSSION

An EMA approach was used to examine levels, patterns, and temporal and contextual predictors of fatigue among individuals with HI and NH. In contrast to Alhanbali et al. (2017) and Hornsby and Kipp (2016), but consistent with Dwyer et al.’s (2019) general fatigue findings, HI and NH groups reported similar levels of fatigue. Diurnal fatigue patterns illustrated increasing fatigue throughout the day for both HI and NH groups, and this reflects patterns found in the wider fatigue literature (e.g., Stone et al. 1996; Kahneman et al. 2004). Within-person situational factors, for example, being in a more or less noisy situation than a person typically reports, or being in conversation with more or less people than usual, had no effect on momentary fatigue. Significant associations were found between person mean listening activity and person mean conversational status and fatigue. However, small effect sizes, the absence of any within-person effects, and the exploratory nature of analyses means that these findings must be accepted cautiously.

There are at least two potential reasons for the unexpected lack of association between hearing group and fatigue. First, comparing a predominantly employed NH group to a largely retired HI group may have masked any potential elevation of fatigue scores among participants with HI, employment versus retired status being associated with higher levels of fatigue (Westerlund et al. 2010). Second, fatigue was self-reported using the BFI “right now” item (momentary fatigue), the full BFI (daily fatigue), and the FAS (trait fatigue); none of which have been validated with a HI sample. The BFI was designed to measure fatigue in cancer patients, and responses in the present study were positively skewed. Alhanbali et al. (2017) and Holman et al. (Reference Note 1) appear to be the only other studies to have used the FAS to measure and compare fatigue in HI and NH samples. In comparison to their median scores, those in the present study were relatively low for both HI and NH groups, even before exclusion of participants with fatiguing health conditions. A potential explanation for this is that the intensive sampling method used here discouraged more fatigued individuals from participating.

Furthermore, general fatigue measures such as the BFI and FAS were not designed to measure listening fatigue and may not be sensitive enough to do so. When used by Alhanbali et al. (2017), the FAS showed that HI groups were more fatigued than the NH control groups, but no correlation was found between degree of hearing loss and fatigue. Hornsby and Kipp (2016) similarly found no such association using the fatigue and vigour subscales of the Profile of Mood States questionnaire (McNair et al. 1971), but they did find that participants with HI were more likely to report severe fatigue compared to normative data. Notably, Dwyer et al. (2019) were unable to show any significant difference in fatigue and vigour levels between cochlear implant users and NH controls using the Profile of Mood States questionnaire. However, a brief listening-related fatigue measure devised by those authors revealed that HI participants were more susceptible to listening fatigue. The present authors were unaware of any widely available, brief listening fatigue measure while designing this study. Dwyer et al.’s three-item listening fatigue scale presents a promising new tool.

In line with Wu and Bentler (2012), Hasan et al. (2014), Wu et al. (2015) and Jensen et al. (2019), participants reported spending most of their time at home, in quiet, nonconversational situations. This suggests that challenging listening situations are either infrequent in everyday life or are missed by EMA surveys. Indeed, qualitative findings suggest that participants in this study were more likely to miss surveys during busier, noisier, or more social situations. In terms of exposure to specific listening situations, participants with NH spent more time in work and more time trying to ignore people speaking in the background, while participants with HI spent more time at home and were less often trying to ignore others in the background. However, when they were, they tended to perceive larger numbers of background speakers to ignore. These differences may be due to group discrepancies in employment status. Another possible explanation is that, compared to individuals with NH, those with hearing loss are more likely to avoid challenging listening situations, and when they do encounter such situations, they perceive background speech to be more problematic. Further research is needed to fully understand these findings.

### Strengths

The EMA method is advantageous in terms of minimizing recall bias and collecting ecologically valid data. This study implemented both signal-contingent and event-contingent sampling, using prompted and unprompted surveys, respectively, to increase the likelihood of capturing listening situations and momentary fatigue as they occurred. At 85%, the response rate was high and comparable to similar research (Galvez et al. 2012, 77%; Henry et al. 2012, 90%; Timmer et al. 2017, 93%). Interview feedback was generally positive, suggesting that the study method was acceptable to participants. Finally, potential covariates and underlying causes of fatigue were measured and controlled for, while participants suffering from fatiguing health conditions were excluded to avoid confounding.

### Limitations

A significant limitation of the present study was the failure to fully match the HI and NH groups based on variables which are pertinent to fatigue, notably age and employment status. The consequences of having a younger and mostly employed control group may have affected the results substantially. Furthermore, all individuals on our participant database have been to National Health Service audiology at some point, indicating a past or present hearing-related issue; the prevalence of tinnitus within the NH group is a particular limitation. The inclusion of both HA and non-HA users in the HI group may have been problematic; future research may consider separating these groups and asking participants to indicate whether or not they are wearing their HAs while responding to questionnaires. In addition, most participants with HI were mild to moderately with hearing loss, which limits the generalizability of the findings and raises the question if fatigue is only an issue for those with severe hearing impairment. Finally, the lower response rate observed among HI individuals potentially indicates difficulty in hearing survey alerts.

## CONCLUSIONS

The present study used an EMA methodology to measure and compare daily-life fatigue between HI and NH individuals and to explore the relationships between challenging listening situations, hearing ability, and fatigue. HI and NH groups reported similar levels and patterns of fatigue, and momentary fatigue was generally unrelated to situational factors. Some evidence was found to suggest that people who spend more time in speech communication/focused listening and conversational situations report higher momentary fatigue, but these findings require further exploration. This study was the first to use EMA to examine fatigue in HI, and thus makes a novel contribution to both hearing research and the wider EMA literature. Moreover, the findings demonstrate the importance of screening for fatiguing health conditions to avoid upwardly biased fatigue scores and loss of sensitivity to the condition of interest. Notable limitations include the use of fatigue measures, which have not been widely used or validated among a HI sample and the prevalence of tinnitus within the NH group. Future research must give careful consideration to fatigue measures, aim to recruit more participants with severe HI and well-matched comparison groups, and consider tinnitus when making hearing group classifications.

## ACKNOWLEDGMENTS

The authors thank Patrick Howell for his assistance in designing and executing the study; Andrew Lavens for providing technical support; and Meigan Thompson for her early work in designing and piloting the study.
